# Between neuroaffirmation and access: Parents’ experiences of autism caregiving in England

**DOI:** 10.1371/journal.pone.0353994

**Published:** 2026-07-17

**Authors:** Anum Farooq, Rachael Mason, Valentina Sclafani, Niko Kargas

**Affiliations:** 1 School of Psychology, Sports Science and Wellbeing, University of Lincoln, Lincoln, Lincolnshire, England, United Kingdom; 2 School of Health and Care Sciences, University of Lincoln, Lincoln, Lincolnshire, England, United Kingdom; 3 Department of Cognitive Sciences, United Arab Emirates University, Al Ain, United Arab Emirates; Father Muller Charitable Institutions, INDIA

## Abstract

Despite growing recognition of the challenges faced by families of autistic children in the United Kingdom, existing research has largely focused on parental stress and individual burden, with comparatively limited qualitative attention to how caregiving demands, service systems, and sociocultural contexts interact to shape family quality of life. Addressing this gap, the present study explored caregivers lived experiences of raising autistic children in England, examining how caregiving demands, access to support, and adaptive responses operate within broader family and systemic contexts to influence family quality of life. Semi-structured interviews were conducted with 12 caregivers of autistic children from diverse ethnic backgrounds, including both White British and ethnic minority backgrounds families, and analysed using reflexive thematic analysis. Six interrelated themes were identified that characterised families’ experiences: (1) The 24/7 Care Ecology; (2) Culture, Religion, and Gender as Contextual Amplifiers (3) Building (and lacking) the Social Support Architecture; (4) Fighting the System(s); (5) Adaptive Resilience and Neuroaffirmation; and (6) Family Quality of Life Consequences. Findings illustrate how intensive and ongoing caregiving demands intersect with fragmented service systems and sociocultural expectations, particularly where cultural, religious, and gendered norms shaped caregiving roles and help-seeking. Notably, neuroaffirmation emerged as a protective and identity-affirming response for some families, reframing caregiving beyond deficit-oriented narratives and contributing to resilience within structurally constrained environments. These findings highlight the need for more accessible, coordinated, and family-centred autism support services that recognise the diverse contexts in which caregiving takes place.

## Introduction

In the United Kingdom, an estimated 700,000 individuals are autistic, with approximately 3 million family members involved in providing care and support [1]. For many caregivers, most commonly parents, caregiving is an all-encompassing and enduring responsibility that shapes daily routines, family dynamics, and long-term life trajectories.

Caregivers play a central role in coordinating support, advocating within health and education systems, and managing children’s diverse needs. However, fulfilling these responsibilities is frequently associated with substantial strain. Research consistently documents elevated levels of stress, anxiety, and depression among caregivers of autistic children compared to parents of non-autistic children [2,3], alongside reduced overall wellbeing [4]. Caregiving has also been linked with social isolation [5], increased physical health concerns [6], relationship strain [7], and significant financial pressures [8] among caregivers of autistic children. Similar challenges including elevated parental stress, social isolation, financial strain and difficulties navigating fragmented health and education systems – have also been documented among caregivers of children with long terms conditions, including cerebral palsy, cancer, diabetes and epilepsy [9,10], as well as among caregivers of individuals with intellectual disabilities across the life course [11]. This suggests that while autism-specific factors shape caregiving in distinctive ways, many of the systemic and relational pressures families face are not unique to autism, pointing to a need for coordinated support structures that address shared caregiving burdens across neurodevelopmental and childhood health conditions. Although this literature has been instrumental in highlighting caregiver burden, it has frequently adopted deficit-oriented frameworks that foreground burden while overlooking positive dimensions such as joy, pride, resilience, strengthened family bonds, and meaning-making [12,13]. Increasingly, caregiving is recognised as a multifaceted experience shaped by both adversity and adaptation; however, these dimensions are rarely examined together.

Family quality of life (FQoL) refers to the overall wellbeing and functioning of the family unit and reflects the dynamic interplay between individual, relational, and contextual influences [14]. Empirical evidence indicates that lower FQoL is associated with younger children with higher support needs [15], co-occurring conditions [16], behavioural challenges [17], and reduced adaptive functioning [18]. Socioeconomic disadvantage including employment disruption, income loss [19], and parental education disparities [20] further compounds vulnerability. In addition, stigma, social judgement, and persistent misconceptions surrounding autism constitute significant external stressors that negatively affect parental wellbeing and broader family functioning [21,22].

Conversely, social support consistently emerges as a critical protective factor in promoting FQoL. Both informal support from friends and extended family and formal support from health, education, and social care professionals can buffer caregiving-related stress and enhance family resilience [16,23]. However, access to such support is uneven, and some families report reduced or withdrawn assistance from extended family networks following an autism diagnosis. Professional support plays a particularly important role in facilitating service navigation, advocacy, and access to appropriate interventions [24,25]. Furthermore, parents who are supported to develop adaptive coping strategies demonstrate more positive psychological and family outcomes [26]. Problem-focused coping, especially when combined with social support–seeking strategies, has been associated with reduced stress and improved quality of life among parents of autistic children [27].

Emerging research has begun to acknowledge that caregiving experiences are not culturally neutral. Sociocultural norms regarding parenting [28], disability [29], gender caregiving roles [30], religion [31,32], and help-seeking shape how families interpret autism, access support, and distribute caregiving responsibilities [33]. In the UK context, ethnic minority backgrounds families may encounter additional barriers, including stigma, linguistic challenges, and systemic inequities in service access [34–36]. However, much of the existing evidence is focused on the perspective of one ethnic group, with limited qualitative insight into how diverse families construct meaning around caregiving, negotiate support systems, and sustain family wellbeing in everyday life. There remains a need for in-depth accounts that centre caregivers lived experiences across diverse backgrounds without reducing them to deficit-based comparisons.

Moreover, dominant narratives of autism caregiving often oscillate between burden-focused discourses [37] and resilience-focused accounts [38], with insufficient attention to how these coexist. Families frequently describe both exhaustion and growth, frustration with services and profound relational meaning. Recent scholarships have also highlighted the role of neuroaffirmative perspectives in reshaping parental understanding of autism, moving away from deficit-oriented framings towards identity-affirming and strengths-based interpretations [39,40]. Yet little is known about how such reframing processes influence family adaptation and overall quality of life.

To address these gaps, the present qualitative study explored caregivers’ lived experiences of raising autistic children in England, with particular attention to how caregiving demands, access to support, and sociocultural contexts interact to shape family quality of life. Its objectives were to explore how families navigate ongoing caregiving ecologies, negotiate formal and informal support systems, and construct adaptive responses within broader structural constraints. While the focus of this study is the experiences of UK caregivers, it is possible that many of the findings, and implications of these, may be transferable to other countries worldwide.

## Methods

### Design

Qualitative design was employed for this component of the study, with the data reported in this paper collected as part of a larger mixed-methods project examining the caregiving experiences of families of autistic children in England. Participants initially took part in a survey study in which they completed demographic questions and standardised questionnaires related to caregiving experiences, social support, coping strategies, and family quality of life. At the end of the survey, participants were invited to indicate their interest in taking part in a follow-up qualitative interview by providing their email address. A mixed-methods approach was employed to capture the complexity and nuance of caregiving experiences, social support, coping strategies, and their impact on family quality of life among caregivers of autistic children. The qualitative component was intended to complement and enrich the quantitative findings by providing deeper insight into the lived experiences behind questionnaire responses, while also allowing exploration of intersectional influences such as ethnicity, culture, gender, and religion on caregiving experiences.

### Ethics

Ethical approval was provided by University of Lincoln Research Ethics Committee (ref: UoL2024_17776). Formal opt-in consent was obtained electronically (via tick-box at the beginning of the survey).

### Community involvement

The research team engaged in consultation with autistic adults, parents/carers of autistic children, and charities/organizations supporting autistic Children and Young People (CYP). They were involved in each stage of research, from design to data collection and analysis, to reporting our findings. This is reported according to the GRIPP-2 short-form in Table 1.

**Table 1:** GRIPP-2 short form for reporting public and patient involvement (PPI) [41].

Section	Overview
Aim	To ensure the study reflected issues of importance to families of autistic children, particularly on domains where exclusion is likely. The aim was to make the study materials, and recruitment process inclusive, and accessible.
Method	Autistic adults, parents/carers of autistic children (we make sure fathers are also represented), and autism charities/organizations that voiced parents (including ethnic minority backgrounds caregivers) were consulted in developing the study. Members reviewed study materials (information sheets, consent form, flyers, interview guides) and provided feedback on recruitment wording, clarity and accessibility.
Outcome	Consultations led to revisions in study documents for clarity. Recruitment materials were adapted with plain, accessible, and inclusive language (e.g., flyers design, language and translations into multiple languages). Flexible participation options (online, in-person) were added. Consultation also informed recruitment networks by identifying trusted community organisations, schools, and parent support groups for outreach specially for ethnic minority backgrounds. Feedback also informed inclusion of new qualitative prompts about stigma, religion, gender roles, and community support.
Discussion	Families’ input ensured that the research questions and analysis captured both the challenges and strengths of caregiving in diverse contexts, ensuring participants lived experiences throughout analysis/writing.
Reflection / Critical Perspective	As a parent carer and a member of an ethnic minority backgrounds community, the PI remained reflexively aware of the importance of representing the lived experiences of all parents, particularly those from ethnic minority backgrounds, and ensured their voices were acknowledged and supported when interpreting challenging accounts.

### Participants

All participants were the informal caregivers of autistic children who were either diagnosed, self-diagnosed, or on the diagnostic assessment pathway. Twelve caregivers were interviewed (*n* = 12), comprising ten female and two male caregivers, all of whom were biological parents living in England. Participants ranged in age from 30 to 46 years, and nine reported that their child had received a formal diagnosis.

Seven caregivers identified as belonging to ethnic minority backgrounds; three from African communities and three from Asian communities, one participant self-described their ethnicity broadly as “BAME” while five identified as White. Ethnicity of the children was not collected, as the study focused specifically on caregivers’ experiences.

Most participants were married (*n* = 10). At the time of interview, five caregivers were official carers, four were employed, two were homemakers and one was unemployed. Seven participants reported caring for a male autistic child, and one caregiver had two autistic children. Table 2 provides further details.

**Table 2:** Participant demographic details.

Participant Number	Age (in Years)	Gender	Relation to the Child	Marital Status	Level of Education achieved	Occupation	Autistic child’s Age (in years)	Child’s Gender	Diagnostic Level/Comorbid conditions	Caregiver Ethnicity
P1		Female	Mother	Never Married	Further education	Employed	5	Boy	Diagnosed	White British
P2	39	Female	Mother	Married	University level	Official carer	7	Boy	On Diagnostic Assessment Pathway	Middle eastern
P3	41	Female	Mother	Divorced	University level	Not currently employed and seeking work	11	Girl	Diagnosed	BAME
P4	36	Female	Mother	Married	University level	Official carer	5	Boy	Diagnosed	South Asian
P5	41	Male	Father	Married	University level	Self employed	8	Boy	Diagnosed	White British
P6	39	Female	Mother	Married	University level	Official carer	8	Boy	Diagnosed (Autism + Epilepsy)	White British
P7	46	Female	Mother	Married	University level	Employed	6	Boy	Diagnosed	Black African
P8	40	Male	Father	Married	University level	Official carer	5	Girl	Diagnosed	White British
P9	40	Female	Mother	Married	_	Homemaker	4	Girl	On Diagnostic Assessment Pathway	Black African
P10	35	Female	Mother	Married	University level	Official carer	5	Girl	Diagnosed (GDD, Autism, eczema, asthma)	South Asian
P11	44	Female	Mother	Married	University level	Self Employed	11	Boy	Diagnosed	White British
P12	30	Female	Mother	Married	Other	Homemaker	6	Girl	On Diagnostic Assessment Pathway	Black African

P, Participant

### Procedure

Following institutional ethical approval, informal caregivers of autistic children were recruited through social media platforms, autism support groups, and community organisations (e.g., schools, libraries, charities, and community centres) using purposive and snowball sampling strategies across England. Participants initially completed an online survey as part of a broader mixed-methods study. The survey was hosted on QuestionPro (https://unioflincoln.questionpro.eu/t/AB3u17lZB3v2ea) and has been available from 30 June 2024 to the present.

The survey could be completed at a time and location convenient for participants. It commenced with a detailed participant information sheet, after which written digital informed consent was obtained. Participants then completed demographic questions and standardized questionnaires. A total of 150 caregivers of autistic children in England completed the survey study. Of these, 15 participants expressed interest in participating in the qualitative interviews. All 15 were contacted and invited to take part in an online or in-person semi-structured interview depending on their availability and preference. Ultimately, 12 participants completed the interviews and were included in the qualitative analysis.

Ethnicity was assessed through self-identification. Participants were asked to select one of two broad categories: “White British” or “Ethnic Minority backgrounds.” This binary categorisation reflects common practice in UK public health and social policy reporting, where disparities are frequently examined across majority and ethnic minority groupings to identify structural inequalities [42]. To mitigate concerns regarding over-simplification and forced categorization [43], participants were additionally provided with an open-ended response option to describe their ethnic background in their own words.

At the conclusion of the survey, participants were invited to indicate their interest in a follow-up qualitative interview by providing contact details. Interviews were subsequently arranged at times convenient to participants, including evenings and weekends, and were conducted either online or in person according to participant preference.

Online interviews were conducted via Microsoft Teams, using available accessibility options (e.g., camera and caption settings, chat). Participants received £10 in recognition of their time, in line with ethical guidance on valuing autistic participants’ expertise and reducing power imbalances [44,45].

Semi‑structured interviews were guided by ten open‑ended questions on caregiving experiences, social support, coping strategies, and broader contextual factors influencing family quality of life and access to services, while allowing participants to shape the discussion. Interviews lasted 60–90 minutes; eleven were conducted online and one in person. All interviews were undertaken by the Principal Investigator, a female PhD researcher. Interviews were audio‑recorded, transcribed verbatim in NVivo, checked for accuracy, and de‑identified before analysis.

#### Analysis.

We conducted a reflexive thematic analysis from a constructionist perspective, attending to semantic and latent meanings. After familiarisation, initial codes were developed through an iterative, interpretive process rather than reliability-oriented; accordingly, inter-coder agreement was not calculated, consistent with the principles of reflexive thematic analysis [46]. To ensure transparency and rigor, the research team maintained a reflexive audit trail, including analytic memos and documented discussions during team meetings, to capture and justify interpretive decisions throughout the analytic process. Through iterative reflexive discussion, we consolidated overlapping ideas and produced six overarching themes and accompanying subthemes that captured the shared meanings across all transcripts.

#### Research Team Positionality.

Given the team’s differing positionalities and disciplinary backgrounds, continuous reflexive practice was embedded throughout analysis. The primary researcher drew on both academic expertise and lived experience as a parent-carer of an autistic child, with her ethnic minority backgrounds informing sensitivity to power, privilege, and cultural nuance; each interview began with a positionality statement acknowledging the limits of cultural understanding and affirming a respectful research space. The wider team contributed complementary neurodivergent, qualitative, social care, and cross-cultural developmental perspectives, with authors identifying as Asian (n = 1) and White (n = 3).

## Findings

**Fig 1:**
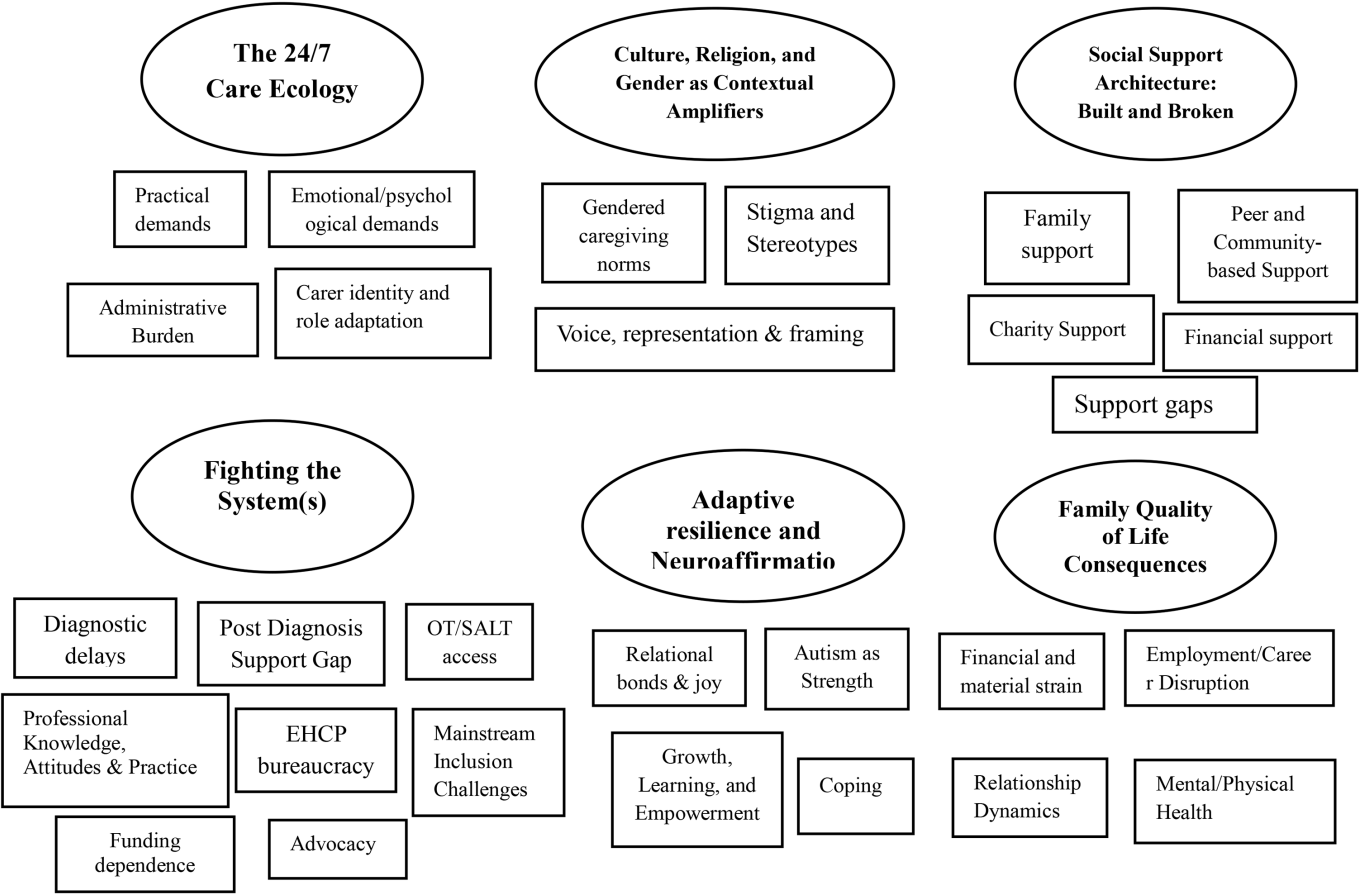
Thematic map showing identified themes and subthemes. The thematic map illustrating caregivers’ experiences of raising autistic children in the England showing six overarching themes (ovals) and associated subthemes (rectangles).

Six interlocking themes characterised families’ experiences: (1) The 24/7 Care Ecology (practical, emotional, and administrative burden plus identity change); (2) Culture, Religion, and Gender as Contextual Amplifiers (gendered caregiving norms, stigma and stereotypes, voice, representation & framing in autism); (3) Building (and Lacking) the Social Support Architecture (family/peer/charity help vs support gaps); (4) Fighting the System(s) (healthcare and education navigation, professional attitudes, resource constraints, advocacy); (5) Adaptive Resilience and Neuroaffirmation (relational bonds, growth, wellbeing practices, coping); and (6) Family Quality-of-Life Consequences (finances, work, health, relationships, social participation). We present each theme with a brief analytic synopsis followed by varied, contrasting extracts. Quotations are attributed by participant ID (P1–P12) to ensure anonymity (Fig 1).

For some families, cultural, religious, and gendered contexts shaped how caregiving responsibilities were understood and enacted, influencing experiences of stigma, support-seeking, and interactions with services. These factors intersected with structural barriers such as language challenges, limited-service accessibility, and underrepresentation within advocacy spaces, contributing to variability in caregiving experiences across the sample. These experiences are captured in the six main themes and differences reported to demonstrate these distinct challenges.

### Theme 1. The 24/7 care ecology

#### Practical demands.

Caregivers described their role as highly demanding, involving continuous supervision, constant availability, and little opportunity for rest. Many highlighted the physical strain of 24/7 care and the vigilance required to manage children’s additional needs.

*“Actually, you’re working probably way more than you would do if you were employed. It’s a 24/7 job, it’s not a nine to five.”* (P6); *“Getting her ready is quite elaborate… eczema creams, steroids, inhaler… certain foods to prevent allergies… this is our biggest burden… you’ve got to be watching her constantly.”* (P10)

#### Emotional/Psychological demands.

Caregiving was described as emotionally overwhelming, marked by persistent stress, anxiety, hypervigilance and concerns about the child’s future. *“I get a lot of anxiety just because they, you don’t feel like you ever get a break from it.”* (P11); *“You look into society for that place where your child would be… is my son ever going to be able to drive? … marry?… get a good job?”* (P7); *“I’m exhausted from the mental load of thinking about everything.”* (P4)

Feelings of guilt and self-blame were common: *“I feel like I’ve failed… with my job and as a mother.”* (P4)

Some ethnic minority backgrounds mothers reported additional gendered concerns: “*I worry more about how she is going to be if I die. Who’s gonna support her? She’s a girl and she’s more vulnerable than a boy.” (P9)*

#### Administrative burden.

Parents described heavy administrative responsibilities when navigating services and completing complex forms. *“I filled out a lot of detail on that form and attached a lot of medical evidence. It took a long time doing it.”* (P10)

#### Carer identity and role adaptation.

Many had taken on the role of full-time carer, often by default, shaped by availability and perceived competence. Some of them expressed it as *“It’s just kind of emerged naturally as of who’s good at what skills.”* (P5); *“It’s just falling into that role due to our abilities to be able to deal with it.” (P6)*

This often occurred alongside unequal division of labour and hidden, unrecognised care work. *“I would say 80%. I take full responsibility for the children…. that increases a mental breakdown for a mother.”* (P12); *“Carers are hidden… at home or in nursing homes, hidden.” (P6)*

### Theme 2. Culture, religion, and gender as contextual amplifiers

#### Gendered caregiving norms.

This subtheme was strongly shaped by cultural norms, particularly among ethnic minority backgrounds participants, who described caregiving as a gendered expectation traditionally assigned to women. These norms positioned caregiving as an inherited female responsibility, accepted without negotiation or consideration of equitable role sharing. *“You know your child and because I did it and my grandmother did it, so you have to do it… that thing of culture thing to them.” (P12).* Another parent-carer said, *“Women are better at doing running the house and men are better at something else.” (P4)*

In contrast, White participants did not typically describe caregiving as culturally prescribed, but rather linked women’s caregiving to perceived innate nurturing qualities. *“You just feel like you are the best person to do it, to do the caring and things... females are more understanding.”* (P11). Even these women acknowledged partner support but maintained that the primary responsibility remained with them. *“For short term he can sort of step in and do stuff … in the next few days I’ll need to pick it up again.”* (P6)

#### Stigma and stereotypes.

Cultural stereotypes and misconceptions about autism shaped caregiving challenges across participants’ accounts. In several ethnic minority backgrounds communities, broad terms such as “handicap” were used for disability, and some languages lacked a specific word for autism, limiting understanding. One caregiver noted, “*We just labeled the children to be handicapped for all…. we do not have a specific word for them*.” (P9) Another parent added, *“In our Indian community… I don’t think they fully understand what autism is.*” (P4)

Caregivers described difficulty explaining autism to older relatives whose views were shaped by contexts where disability was rarely discussed: *“…. just my parents do not really understand it and not really believing it’s a thing.”* (P11) Concerns were frequently minimised, with family members suggesting parents were “worrying too much” or that children would “grow out of it.” One caregiver recalled, “Before K** got a diagnosis… you shouldn’t say he’s autistic… that kind of minimizing.” (P4)

Invalidation also occurred within households, where caregiving observations were dismissed as exaggeration: “*Being alone with her at home, maybe I’m just exaggerating and making things.”* (P9) Extended family members often attributed behaviours to poor discipline, assuming intentional control: *“You need to put your foot down more.”* (P7)

Media-driven stereotypes further shaped expectations by masking the range of autistic experiences: *“All the autistic people you often see on TV are quite high functioning… I tend to say she has developmental delays because I feel like maybe that better gets across.”* (P10)

Stigma surrounding invisible disabilities heightened fear of judgement and concealment. One caregiver shared, “*I don’t openly advertise myself as autistic to others because I fear that I might be judged differently than normal.”* (P5) Another noted increased public scrutiny compared to visible disabilities: *“You can tell people are looking at you like something’s wrong with that kid… if she was in a wheelchair or had a physical disability there would be a lot less judgment in publi*c.” (P8)

Many caregivers struggled to express emotional distress due to stigma*: “I struggle to talk about it… ‘every parent has difficulties.’”* (P4); *“You’ve got to be able to express that without fear of, ‘you shouldn’t be saying that about your child.’”* (P6)

For ethnic minority backgrounds caregivers, stigma was intensified by cultural denial, delaying acceptance and support: *“First of all, my husband stays in denial.”* (P12); *“They really didn’t want to accept anything.”* (P10). This left carers feeling isolated and underrepresented in support spaces. *“I access support groups or coffee mornings for Special Education Needs and Disability* *(SEND) families, but they are less represented by other minorities… there’s more stigma within ethnic minorities.”* (P4)

#### Voice, representation and framing in autism.

Parents raised concerns about the limited representation of their voices within autism advocacy, particularly among carers of children with high support needs, co-occurring conditions, and limited capacity for self-advocacy. *“I have to advocate… when you hear about autistic issues, you’re hearing from those who can speak and communicate for themselves… not from autistic people with learning disabilities or those who need 24/7 care… that’s what’s missing when parents of children with significant needs are overlooked.” (P10)*

Language policing emerged as a prominent theme, especially among caregivers from ethnic minority backgrounds. Participants described how evolving neurodiversity-related terminology created additional pressure and anxiety. *“There’s a cultural war going on with autism at the moment… very much about policing language… I experienced a lot of pushbacks against even using word phrases like higher needs or lower needs.” (P4)*

For parents whose first language is not English, these expectations were particularly distressing. *“She’s trying to express herself… a vulnerable mother asking for support… and you ignore that and spend the whole time lecturing her about using the word ‘normal’.” (P10)*

Fear of community backlash further deterred some ethnic minority backgrounds caregivers from discussing early interventions or support options. Even brief mentions of such interventions often triggered intense backlash. *“I went on a UK support group and innocently asked why no autism charity campaigns for early intervention… and I got such pushback. People said I was a child abuser, that I wanted to take away my child’s autism and make her neurotypical and I was this evil monster, and it was awful.” (P10)*

Among Muslim caregivers, disability was frequently framed as a divine test or blessing, reinforcing acceptance and meaning making within caregiving roles. As these caregivers expressed, *“I’m blessed because it’s a reward.”* (P9); *“It’s in our religion, whatever has been given to you it has been given you to use for a reason.”* (P12)

### Theme 3. Social support architecture: Built and broken

#### DLA and financial support.

Access to financial support through disability-related benefits, such as Disability Living Allowance (DLA) and Carer’s Allowance, emerged as an important source of relief for many parents, as reflected in one parent’s account: *“It does help because with the DLA you can get into a lot of places as a carer for free, so that does help.” (P11)*

Several participants noted that the financial assistance and related exemptions such as being released from work-related requirements under Universal Credit allowed them greater flexibility and time to care for their child: *“It makes my life more flexible and has more downtime than if I was expected to seek work. I’m exempt from Universal Credit work requirements because she received DLA.” (P3)*

However, despite recognising these supports, parents also expressed concerns about the adequacy and fairness of the current benefit system. “H** is profoundly disabled, so he should get more… somebody that’s milder… you could skew them with slightly less money.” (P6)

Others voiced frustration about broader policy changes and cuts to disability benefits, particularly in the context of economic austerity: *“I know obviously the country is in a lot of financial difficulty at the moment, but they’re now attacking all the disabled benefits.” (P6).*

#### Family support.

Family emerged as a crucial source of informal support, providing both practical assistance and emotional relief for caregivers of autistic children. Multiple parent-carer accounts of these supports is: *“My husband does a lot of his care, from washing him every morning to getting him ready.”* (P7); *“He’ll have a sleepover at his grandparents’ house every few months.”* (P4); *“Her sisters push her a lot like motivating her to do things that she has never done before.”* (P12)

Despite the benefits of family support, several parents also acknowledged that support networks can be inconsistent, often depending on proximity, child’s support needs, or family understanding of carers roles. *“Not much support really. None of her grandparents live locally.”* (P3); “*They try a little bit, but it’s nowhere close to the same level they would give if she didn’t have these needs.”* (P10)

#### Peer and community-based support.

Many parent carers highlighted the value of connecting with other parents through both formal and informal peer networks. Online groups and social media platforms were accessible sources of information, advice, and emotional support. *“I get a lot of help on Facebook.”* (P1); *“I’ve joined the WhatsApp group of the autism carer for outings, activities, events and information.”* (P7)

Parents described these spaces as offering comfort through shared lived experiences and practical guidance on navigating services and benefits. *“It’s nice to be in a room with other parents who are kind of going through the same thing as you and you can learn from each other.” (P1)*

Some parents engaged in structured courses or local support groups that offered learning opportunities and a sense of belonging. *“I learned all this with the courses… with the connection* *that I have with people… to ask other parents how they are dealing, what kind of support there’s there.”* (P9)

#### Charity support.

Charities serve as one of the most significant formal sources of support that carers rely on for advocacy and assistance in navigating the bureaucratic processes involved in applying for DLA and completing Education, Health and Care Plan (EHCP) forms. *“I got a lot of help from the charity; they know a lot about the law when it comes to providing education for special educational needs and the tribunal process.” (P11)*

Other expressed satisfaction with the charity support in accessing other related services, information, and signposting*. “We’ve got a lot of knowledge and signposting on things that are happening.” (P4)*

They often provided respite care, holiday club and related services at affordable rates which would otherwise be difficult for many parents to afford. *“Help us have a break as well … quite reasonable…They keep the costs quite low.” (P11)*

#### Support gaps.

Carers consistently reported substantial gaps in support and service provision for autistic children with significant support needs. A prevailing “one-size-fits-all” model often excluded children requiring intensive support, limiting access to childcare, holiday clubs, and extracurricular activities due to insufficient staff training and capacity. As one parent noted, *“it’s really hard to find him like a holiday club provision because his needs are high”* (P1). Another explained that limited communication skills *“instantly rules out a large chunk of services.”* (P8)

Even when respite care existed, it was described as limited, inconsistent, and subject to long waiting lists: *“She’s been trying to get it for at least a year now, and they haven’t even assessed for it yet”* (P6).

Scarcity of specialist and culturally responsive services further restricted access, particularly for ethnic minority backgrounds families. *“They’re less likely to go to a support group where there’s going to be no other ethnic minorities because they’re not going to be talking about religion and God.” (P4)*

Navigating the fragmented UK care system required strong advocacy skills, disadvantaging families with language or cultural barriers. *“I’m imagining if a mother who doesn’t perhaps speak English… to even be able to ask the right questions or go online and search.” (P7)*

### Theme 4. Fighting the system(s)

#### Diagnostic delays.

A major challenge reported by parents was significant delay in securing an autism diagnosis. The process was described as lengthy and fragmented, involving multiple professionals and prolonged waiting lists. *“It’s a very, very long process… dragging, waiting list nonstop”* (P9). Even initial assessments could take years: *“24 months is the waiting time to meet the specialist”* (P2). Systemic backlogs intensified delays: *“Our London borough has such a backlog of autism cases”* (P10). Some families pursued private assessments to bypass NHS waits, but these were not always accepted, requiring repeat assessments: *“He did have to have a diagnosis with [the NHS] as well”* (P11).

#### Post diagnosis support gap.

Across interviews, participants highlighted a lack of post-diagnostic guidance and structured pathways describing the process as isolating and confusing. *“They don’t tell you where to go… you’re on your own. You have to do the research”* (P7). Another parent asked, *“The real sticking point is straight from diagnosis to what do I do next?”* (P8).

Many children were diagnosed only after starting school, at which point families were discharged from community services and redirected to school-based support. *“They just discharged him basically… because he was now going into school”* (P4).

#### Occupational therapy (OT)/speech and language therapy (SALT) access.

SALT and OT were the most sought-after NHS services, yet access to OT was often limited by long waiting times. Multiple accounts of parents were *“There is no provision for OT in the NHS for sensory… they send us just a video to watch”* (P4); *“Even with the support of the autism diagnosis team, it took three attempts to get her on the OT waiting list.”* (P3)

Limited access to SALT and long waiting times were major parental concerns as a mother said, *“He sees a SALT at school… probably once every three months”* (P1). Another expressed her concern, *“It was still such a long battle to get support, and especially to get speech and language support.”* (P10)

Due to the restricted availability of SALT services, therapists often conduct only brief observations, which are then used to set goals; however, no consistent follow-up sessions take place in schools. *“They would like to see him in this classroom or in the nursery for like an hour and then write a report with targets… they never really had any sessions with him.”* (P4)

#### Professional knowledge, attitudes and practice.

Carers reported that healthcare professionals often dismissed their concerns or demanded excessive evidence, leaving them feeling mistrusted, stressed, and unsure where to seek support. *“I said he’s not talking and she was like saying, oh, he will, like trying to brush it under the carpet.”* (P1); *“I took both my boys to the GP, and they said we need more evidence so, they basically didn’t believe me.”* (P11)

Parents perceived healthcare professionals as delaying assessments and relying on brief observations. *“They will see a child for two days or maybe one day… and they will make a decision”* (P12). Professionals were also viewed as lacking practical experience with complex needs: *“They’re not geared towards the really severe, profound autism”* (P6). Another parent added, *“The GP, they don’t have any idea”* (P2).

Carers reported challenges with education professionals, often feeling their concerns were dismissed. “The SENCo said F** has no issue…I said she’s not just quiet, she cannot communicate” (P12). Limited autism training among school staff was widely noted: *“The teaching assistants probably have one hour of training”* (P4); *“The teachers might have had a few days in their PGCE, but they’ve not actually had sustained, specialist experience or training in autism”* (P10). Limited time for parent–teacher communication further constrained collaboration: *“They have no time to meet parents frequently”* (P2).

In contrast, many parents praised the expertise and skills of staff in specialist schools, emphasising that these professionals were better equipped to provide appropriate educational support: *“The fact that her teacher looks after special needs kids day in, day out — she’s used to adapting the curriculum for them, knows how to teach, engage, and help them, and all the assistants working there have that long experience and training.”* (P10)

#### EHCP bureaucracy.

Parents highlighted the EHCP as the primary gateway to school-based support; without it, access was severely limited. *“If you do not have EHCP plan, your child cannot be taken full time”* (P9). The application process was described as bureaucratic and time-consuming, often leaving parents to complete it themselves: *“The EHCP process was a lot… a lot of assessments”* (P4), with waits of *“like six months for the EHCP to go through”* (P6). Challenges continued post-approval, as plans were seen as misaligned with children’s needs due to limited professional assessment: *“She’d never met an educational psychologist… the EHCP was miles off”* (P8).

#### Mainstream inclusion challenges.

Most parents reported that mainstream schools lacked adequate support, with children frequently isolated or insufficiently supervised. *“They put him in one room alone—it was not better for him”* (P2). Staff limitations were evident: *“If you have more severe needs, there is very little we can do in a mainstream primary school … beyond babysitting”* (P10). Parents also described unsafe practices, including children left unattended: *“I found her standing in the corridor, just abandoned there… for 20–30 minutes”* (P12).

Even when accepted into mainstream settings, parents reported frequent calls to collect their children for minor incidents, disrupting employment and routines: *“Because it’s not a special needs school, they can’t tolerate anything”* (P9). Choosing between mainstream and specialist provision was described as an ongoing struggle, with long delays despite EHCPs: *“We’ve been trying to move schools since reception”* (P1). Parents expressed frustration at being pressured by local authorities to remain in mainstream placements without adequate resources: *“They need to provide the support and stop complaining”* (P11).

#### Funding Dependence.

Parents linked difficulties navigating healthcare and education systems to inadequate government funding for SEND services. *“There’s just not enough money… with special needs budgets”* (P10). School staffing and provision were described as funding-dependent: “H*** special needs school does not have breakfast clubs… because they don’t have the funding for the extra level of staff” (P10). Another parent emphasised the need for financial investment to enable inclusion: *“They need to be able to provide the money so that the schools can put the support in place”* (P11).

#### Advocacy.

Advocacy emerged as central to navigating education and healthcare systems, as support was rarely automatic or coordinated. Parents described the need to push persistently: *“You have to fight… you have to advocate for your child”* (P7); *“If you can advocate… your child will get more support”* (P4). In healthcare, advocacy was required to secure referrals, medication, and ongoing treatment, which was *“dependent on me advocating and pushing for it”* (P10). Medication access was described as an ongoing struggle: *“I have to fight every three months”* (P9).

Within education, advocacy was continuous and exhausting, extending to appeals and specialist placements: *“It just feels like a constant fight all the time”* (P11). When schools failed to initiate support, parents completed EHCP applications themselves: *“I basically did a parent submission”* (P6), underscoring advocacy as essential for accessing provision.

### Theme 5. Adaptive resilience and neuroaffirmation

#### Relational bonds and joy.

Parents’ caregiving was supported by the strong emotional bond they share with their autistic children, which provided a key source of joy, fulfilment, and meaning. *“I’m so close to them and I work with them every day. It’s just ingrained into me all the knowledge and things, so we are very close.”* (P11)

Their connection offered emotional relief and strengthened their sense of purpose. *“However heavily your day is, you still find the joys of being a mom to him”* (P7); “*I tweaked her nose and said, ‘honk honk,’ and she burst into laughter and ran off saying it back.”* (P8).

#### Autism as strength.

Parents demonstrated notable resilience and adaptability, often reframing autism as a source of strength rather than a limitation. Autistic parents, in particular, described having unique insight into their children’s needs due to shared lived experiences, enabling them to provide more empathetic and attuned support. *“There’s so much potential untapped if we could free up all of these sort of like daily skills then autistic children could tap into so much like growth and opportunity.”* (P5). Another autistic mother expressed: *“I think also being autistic and ADHD myself, I get I have a quite a large insight as well into how they’re feeling.”* (P11)

Parents consistently emphasised that it is the environment not autistic individuals that must adapt. “*It’s not the autistic person who’s broken; it’s the world that’s not set up for the autistic person.”* (P5)

#### Growth, learning, and empowerment.

Caregiving fostered substantial personal growth, with caregivers reporting increased patience, resilience, emotional maturity, and transformative impacts on their life trajectories. *“And now I am more resilient.”* (P3), *“I have now more tolerance.”* (P2). One parent shared, *“I wouldn’t be at university if it wasn’t for her… if Uni goes well there’s a reasonable chance, I could end up doing something in additional needs space.”* (P8)

Caregiving also deepened parents’ self-awareness. *“He’s teaching us to reflect; be self-aware. It taught us to take a step back to really learn about the way we do things typically.”* (P4). This reflective process led some parents to seek an autism diagnosis for themselves: *“You start making sense of yourself with an autism diagnosis… ‘oh, that makes much more sense.”* (P8)

Parents also described gaining broader life perspective: *“It has given me real insight into life as a whole”* (P6); another parent shared, *“He’s taught us a slower-paced life.”* (P4).

This growth often motivated parents to support others, contributing to community reciprocity: *“I can give them from my experience… I can support them”* (P2).

#### Coping.

Social support emerged as a central coping mechanism, with family, friends, and peer groups offering emotional relief and practical help. As one parent explained, *“When my mood plummets, I go to my mum or sister… it lifts my mood enough to keep going”* (P6). Online groups also provided guidance and shared experience: *“You get info, support, and advice… it helps you cope.”* (P10).

Parents used a range of practical wellbeing strategies, such as: *“A break as a family helps recharge your batteries.”* (P2); *“Meditating helps… getting some time away.”* (P4). Emotional release was also an important coping mechanism: *“You have to let yourself freely cry… just to let it out.”* (P12)

Positive emotions, such as hope, gratitude, and love were key sources of coping. One parent expressed gratitude: *“Alhamdulillah… I’m blessed with four wonderful children”* (P9). Children’s progress fostered hope: *“He’s starting to hold a pen… that gives us hope”* (P4). Affection from children also sustained parents: *“When he hugs you and tells you everything is going to be alright.”* (P7)

Optimism about children’s future development further strengthened coping: *“She has outgrown many things… she has made a lot of improvement”* (P12)

Religious coping was described exclusively by Muslim parents, who viewed faith as a major source of strength and acceptance: *“My religion has been a pivotal part of helping me achieve where I am today.”* (P7)

### Theme 6. Family quality of life consequences

#### Financial and material strain.

Caring for an autistic child substantially affected families’ financial wellbeing, with many households reduced to a single income due to full-time caregiving responsibilities. *“Our financial security has changed because one of us has had to give up their job.”* (P4). Another parent echoed this concern: *“We’re one wage down unfortunately, and even with the disability and the carers allowance, it probably doesn’t make up for that extra wage we could be earning.”* (P11)

Parents also reported increased daily living expenditure, particularly for children with high support needs. *“He goes through huge amounts of nappies, wipes, he breaks things, he throws* *food all over the floor… And then you’ve got equipment to buy for him like his pram. You’ve got to drive more places because he can’t walk, so that costs petrol money.”* (P6)

#### Employment/career disruption.

Most carers described significant disruption to their employment, education, or long-term career aspirations. Participants expressed a sense of loss, limitation, and sacrifice linked to their caregiving role: *“If I wasn’t a carer, I’d definitely probably have a career.”* (P11); *“Before we realised the level of her needs, I was hoping to have a career in academia… That’s not going to work with my daughter… So, it really restricts my career options.”* (P10)

Some parents who attempted to continue education or employment reported that institutions showed limited flexibility. *“There are times when work backs up because other things matter more… I regularly get attendance texts from the university, which I ignore.”* (P8)

Traditional full-time work was often incompatible with caregiving demands: *“I can’t work after three… I always have to make sure I’m there to pick them up every day.”* (P11)

Parents who remained in employment emphasised how essential supportive employers were: *Because I’ve always sort of been in academic circle, within that sort of academic circle it’s quite good. I’ve had flexibility in previous jobs.”* (P5)

#### Relationship dynamics.

**Marital Strain:** All participants reported that caregiving placed pressure on their relationships. The constant demands left limited time or emotional capacity for partners to connect. *“We occasionally have a date night… literally day-to-day you get zero time together.”* (P5); *“It’s been an extreme strain on us… at times we’ve barely been able to keep going.” (P6)*

However, a few carers reported that the shared struggle strengthened their relational bond. One participant described how facing challenges together fostered greater closeness and mutual support: *“When you have a problem, you become closer and more supportive because we have something in common. We have to raise this together. I can’t do it by myself, and he can’t do it by himself. Together, we have to lift it up.”* (P2)

**Sibling Dynamics:** Siblings were often described as playing a supportive role, contributing positively to their autistic brother or sister’s development: *“Her sisters push her a lot… She learns from them, she learns words, sentences…” (P12)*

Parents frequently perceived siblings as more empathetic and mature because of these experiences: *“I think it’s made her more caring and understanding that everybody’s different.” (P1)*

However, many carers reported difficulty spending quality time with their non-autistic children: *“We tend to do things separately… My wife took the eldest away… I stayed home with both small people.” (P8)*

Parents described making conscious efforts to maintain balance: *“I try to be conscious of that… I try to distribute time and encourage the children to speak to me.” (P7)*

#### Mental/physical health.

Caregiving placed a considerable emotional burden on parents. Many reported persistent stress, isolation, and low mood: *“It definitely has a massive toll… more of a depression-type problem caused by the kids’ situation.”* (P6); *“You’re on your own a lot of the time, which can affect your mental health.”* (P11)

Several carers highlighted the need for empathetic emotional support: *“Having some sympathy and understanding… someone who says, ‘I’m sorry, this must be really hard for you.’”* (P10)

Parents also described physical strain from manual caregiving tasks: *“He’s a big boy… I tried to carry him but now I have pain in my back.”* (P2); *“I’ve damaged my back now… the physio thinks it’s directly related to my caring role.”* (P6)

Sleep deprivation was a persistent challenge due to their child’s irregular sleep patterns: *“She’ll wake up in the middle of the night and be up for hours… I’m often up with her. Very sleep deprived… it’s affected me physically in lots of ways.”* (P10)

#### Reduced social participation.

Many carers reported a significant reduction in social participation, often resulting in feelings of withdrawal and isolation: *“You lose people… friends you had for years… you’re on your own.”* (P12)

Experiences of social judgement further discouraged participation: *“We don’t go to families or friends because we feel judged the whole time.”* (P11)

Some avoided social settings due to concerns about their child’s behaviour and others’ reactions: *“I couldn’t take him there because he’d smash things… your child’s going to be like pulling the clothes off, peeing on the floor.”* (P6)

## Discussion

Our study highlights how parents of autistic children navigate intensive caregiving demands and emotional labour within fragmented health, education, and social care systems. Extending existing literature, the findings show that parental stress, uncertainty, and advocacy are shaped by the interaction of caregiving responsibilities, social support availability, and broader contextual influences. Within these under-resourced systems, adaptive resilience and neuroaffirmative meaning making develop unevenly, contributing to disparities in family wellbeing.

Consistent with prior work, parents reported intense supervision demands, emotional strain, persistent anxiety, and cognitive overload [47,48]. These pressures were exacerbated by the administrative weight of navigating health, education, and welfare systems. Caregiving remained heavily gendered, with mothers especially from ethnic minority backgrounds positioned as default carers through longstanding cultural norms that emphasised self-sacrifice and domestic responsibility [30]. The predominance of mothers in the sample (10 of 12) reflects this pattern directly, with caregiving roles rarely discussed or negotiated within the family context regardless of ethnicity, though the expectation was particularly pronounced among ethnic minority backgrounds participants (P4, P10, P12). This intensification of caregiving labour mirrors wider evidence on gendered caregiving inequalities, and is further compounded by employment disruption: eight of twelve participants were not in paid employment at the time of interview, including those identifying as official carers or homemakers, illustrating concretely how caregiving demands constrain labour market participation, a point returned to in Theme 6.Stigma emerged as a pervasive and universal experience [21]. Parents described the challenges of invisible disabilities, lack of public understanding, and affiliate stigma that shaped identity, distress, and social withdrawal [22]. Among ethnic minority backgrounds families, stigma was deepened by the absence of culturally meaningful terminology for autism [34], reliance on pejorative descriptors, and intergenerational tensions with relatives who denied or reframed the child’s needs [49]. Shame and denial are also acted as barriers to help-seeking in these families [34,35]. Narrow media portrayals of autism as exceptional ability further fueled misunderstanding, unrealistic expectations, and reluctance to disclose struggles [50]. These dynamics were particularly evident among participants whose children had co-occurring conditions (P6: autism and epilepsy; P10: global developmental delay, autism, eczema, and asthma), who described the compounded stigma of navigating both autism-related misunderstanding and the visibility of their children’s additional medical needs, and who consistently reported the most complex and under-resourced caregiving contexts across the sample.

A novel contribution of this study is its examination of how parents engage with neuroaffirmative discourse. Many embraced autism as difference rather than deficit and valued their child’s unique ways of thinking [51]. Autistic parents used lived experience to create validating environments [40]. However, access to neuroaffirmative practices was not equally distributed. Several ethnic minority backgrounds parents felt judged when using culturally familiar terms or when lacking fluency with advocacy language, creating additional emotional labour, cultural displacement and barriers in accessing support. Recent scholarship has highlighted that the neurodiversity movement, while progressive in intent, remains rooted in Western, Global-Northern frameworks that may not translate seamlessly across cultural and linguistic contexts [52]. These findings highlight how neuroaffirmation risks becoming exclusionary if used as a linguistic or ideological gatekeeping tool, an issue reflected in broader concerns about whose voices shape autism discourse and policy [53,54]. Together, these findings underscore the need for culturally responsive, decolonial approaches to neuroaffirmation that preserve its core values while accommodating diverse ways of knowing, communicating, and seeking support.

Systemic barriers were a major source of strain for families. Parents described long waiting lists, fragmented services, minimal post-diagnostic support, and poor signposting [55]. Participants also highlighted barriers experienced during the pre-diagnostic and waiting period, including restricted access to services without a formal diagnosis, uncertainty surrounding support pathways, emotional distress, and the additional caregiving burden associated with prolonged waiting times. Although the interview schedule did not specifically include direct questions regarding diagnostic waiting pathways, these experiences emerged organically and consistently throughout participants’ narratives.

Families of children with significant support needs, including multiple diagnoses, intellectual disability, physical health conditions, minimally speaking autistic children, reported that they often receive the least support. The families expressed their concern in terms of provider inexperience in understanding the needs of these children or expressed the concern that they might not understand the realities of additional needs. Although participants highlighted the need for greater professional training and awareness, these experiences may not solely reflect provider lack of knowledge or expertise but could also relate to broader systemic and commissioning constraints affecting autism service provision, including limited resources, staffing pressures, poor care coordination, limited training, and variability in local service availability [56], despite professionals’ intentions to provide more comprehensive care.

An important point highlighted in the findings was that, although children with additional and more complex support needs often experienced relatively quicker access to diagnostic assessment pathways, ongoing support and service provision frequently remained insufficient to meet families’ caregiving needs. For example, one parent-carer of a child with autism, global developmental delay, eczema, and asthma described significant challenges in accessing appropriate childcare support. The parents explained that they were unable to secure a nursery placement for their child until the age of five due to the child’s extensive medical and support needs. The parents also described the demanding daily caregiving routine associated with managing the child’s eczema and asthma, particularly during morning preparations, which added further strain to the caregiving experience.In terms of advocacy, these families reported under-representation within autism advocacy spaces [57]. Advocacy becomes a further challenge for those who navigate services in a second language and encounter cultural narratives with limited conceptual space for autism [58].

Overstretched speech and language therapy and occupational therapy services, with tens of thousands of children on waiting lists, continue to reinforce inequities in access to care [59,60]. Parents reported that children with lower or moderate support needs are often excluded as services prioritise those with higher needs, resulting in delayed or absent intervention that undermines child outcomes and intensifies parental stress. Parents’ dissatisfaction with Occupational Therapy (OT) and Speech and Language Therapy (SALT) services may partly reflect a mismatch between parental expectations and the current scope of these services, which often focus on assessment, recommendations, and supporting implementation through schools or caregivers rather than providing ongoing direct intervention. The findings also highlight the potential role of communication gaps regarding what families can realistically expect from these services, including limited explanation of service pathways, lack of transparency around assessment and diagnostic decision-making processes, and uncertainty regarding the nature and availability of professional support. These findings emphasise the importance of clearer professional communication, guidance, and signposting to better support families navigating these services, increasing awareness and understanding to better inform expectations. In addition to structural gaps, parents described dismissive professional attitudes, including medical gaslighting and limited understanding of neurodivergent needs, which further eroded trust in services and compounded emotional burden [61,62].

Education challenges such as limited staff training, inconsistent support, bureaucratic EHCP processes and limited specialist schools further undermined children’s progress and increased parental advocacy burdens [34,63,64]. This highlights the need for streamlined pathways, such as one-stop SEND navigation hubs, advocacy brokerage, and personalised support across educational settings. Professional training in autism- and culture-informed competencies for clinicians and educators remains essential.

Social support functioned as an important buffer [47,65]. Emotional, practical, and respite support from family and friends was beneficial, though unevenly distributed and often constrained by stigma or misunderstanding [66]. Peer support through WhatsApp, Facebook, and voluntary organisations was particularly valued for its accessibility and lived-experience guidance [67]. These findings highlight that parents of autistic children need diverse formal and informal support from multiple systems.

Parents demonstrated substantial adaptive resilience, characterised by strong emotional bonds, shared meaningful activities, and active engagement with training, information, and community resources that reinforced coping [51,68]. Consistent with literature that resilience was reflected in parents’ ability to persevere toward a sense of normalcy, establish sustainable caregiving routines, and shift from attempts to “fix” their child toward acceptance and adjustment [38]. For some parents, caregiving also prompted reflection on their own neurodivergence, leading to self-exploration and, in some cases, later-life diagnoses [39,69]. Faith played a distinctive role among Muslim caregivers, offering acceptance and meaning that supported coping [31]. Thus, neuroaffirming approaches developed in partnership with trusted community or faith leaders, supported by translated and accessible guidance will help these communities.

Caregiving demands significantly affected financial stability, employment, relationships, and health, consistent with prior research [19,70]. In the present study, many mothers reported leaving paid employment due to limited school provision and inadequate service support, resulting in substantial financial strain. Most parents highlighted the impact of having one parent fully engaged in caregiving on overall family well-being [71]. Although financial benefits such as Disability Living Allowance (DLA) and Carer’s Allowance offered some relief, they were widely perceived as insufficient to meet the additional costs associated with complex needs, indicating a need for more targeted financial support policies.

Partner relationships were often strained by stress and reduced shared time [72]; however, some couples reported strengthened bonds through shared struggles, describing their family as a united team working together to protect and enhance their well-being [73]. Sibling relationships similarly reflected both warmth and conflict [74]. In addition, caregivers reported chronic stress, sleep disruption, fatigue, and depressive symptoms [75] emphasise the cumulative impact of caregiving and systemic shortcomings. Our findings suggest that these difficulties are not only a consequence of caregiving demands but also of the cumulative strain created by navigating fragmented services, limited support, and ongoing uncertainty.

Several limitations should be considered when interpreting these findings. Recruitment through community organisations, charities, and online networks may have resulted in a sample of caregivers who were relatively engaged with support systems, potentially underrepresenting more isolated or digitally excluded families. Data were collected exclusively in English, which may have limited participation from families with limited English proficiency and constrained the depth of culturally nuanced insights. The study captured the perspectives of primary caregivers only. Partners’ experiences and contributions to caregiving were not independently assessed, and participants were asked to reflect on their own experiences rather than those of their partners. Future research would benefit from including dyadic or multi-perspective designs to capture how caregiving responsibilities and experiences are distributed and negotiated within couples. The experiences were not analysed separately according to the families’ stage within the autism diagnostic assessment pathway (e.g., awaiting assessment, undergoing assessment, or post-diagnosis). Families at different stages may experience distinct challenges related to uncertainty, access to support, advocacy burden, and service navigation. Due to the relatively small qualitative sample size, subgroup comparisons were not feasible within the current study. Future research could further explore how caregiving experiences differ across stages of the diagnostic assessment process. Moreover, future research should integrate neurodiversity-affirming frameworks with culturally grounded epistemologies to better reflect relational understandings of disability and address the marginalisation of ethnic minority backgrounds communities.

## Conclusion

Taken together, these findings underscore the complexity of caregiving across familial, systemic, and contextual settings. Families faced substantial demands related to intensive caregiving and persistent difficulties accessing timely, coordinated, and appropriate services, while simultaneously demonstrating resilience, advocacy, and adaptive capacity in navigating support systems. The study further highlights that access to neuroaffirmative understandings and practices is uneven and shaped by broader structural and contextual factors, including how services engage with diverse families. Without culturally inclusive and accessible pathways, neuroaffirmative approaches risk reinforcing existing inequities.

Overall, the findings emphasise the urgent need for family-centred, culturally sensitive interventions, equitable support pathways, and coordinated, adequately resourced health, education, and social care systems that recognise structural inequalities and contextual influences on caregiving. Parental resilience, while admirable, should not be treated as a substitute for adequate systemic support.
